# Heat and carbon monoxide exposure: Is two better than one?

**DOI:** 10.1113/EP092198

**Published:** 2024-08-22

**Authors:** Kevin L. Webb, José González‐Alonso

**Affiliations:** ^1^ Department of Cardiovascular Medicine Mayo Clinic Rochester Minnesota USA; ^2^ Sport, Health and Exercise Sciences, Department of Life Sciences, College of Health, Medicine and Life Sciences Brunel University London Uxbridge UK

**Keywords:** aerobic performance, erythropoietin, heat acclimation, renal oxygenation

1

A fundamental aim in the field of human physiology is to understand and delineate the limits of human function. In the realm of exercise physiology, sports and aerobic performance, the preceding decades have been marked by investigation into exogenous mechanisms of blood doping, exemplified by the administration of recombinant erythropoietin (EPO), erythropoiesis stimulating agents and whole blood transfusion. Yet, the line between such ‘artificial’ methods of blood doping and natural adaptation becomes blurred as we better understand how to manipulate haematological parameters through methodologies currently allowed by international sporting committees.

In this issue of *Experimental Physiology*, DiMarco et al. provide insight into two acute interventions that elicit haematological adaptation with potential ergogenic effects (DiMarco et al., 2024). First, carbon monoxide exposure reduces arterial blood oxygenation, impairing oxygen delivery to the visceral organs such as the kidneys with little renal blood flow compensation (Schmidt et al., 2020). In response, the kidney detects a reduction in oxygen delivery and stimulates the production of EPO, increasing haemoglobin mass in compensation (Montero & Lundby, 2019). Through differing mechanisms, acute heat exposure is generally considered to reduce renal blood flow, lowering oxygen delivery and thus evoking a similar EPO response in compensation (Oberholzer et al., 2019). The potential utility of carbon monoxide and heat exposure is to increase the total amount of red blood cells and haemoglobin mass, which have been positively associated with a greater aerobic capacity and athletic performance (Schmidt & Prommer, 2010). Both methodologies elicit haematological responses that have been studied previously, but the novelty of DiMarco et al.’s work stems from (1) evaluating a hypothetical potentiating effect of performing acute carbon monoxide exposure and passive heating concomitantly, and (2) investigating potential sex differences yet to be addressed.

DiMarco et al. undertook their investigation with 16 participants (eight males and females), measuring the circulatory EPO response and appropriate physiological parameters during three randomized visits (carbon monoxide inhalation, heat exposure through hot water immersion, and both carbon monoxide inhalation and heat exposure) (DiMarco et al., 2024). Each visit involved 6 h of venous measurements following the acute intervention. For the visits containing carbon monoxide exposure, a single bolus was rebreathed for 10 min with the aim of increasing carboxyhaemoglobin to 10–15%. For the visits with hot water immersion, participants sat upright in heated water (40°C) for 45 min while measuring core body temperature through an ingested pill sensor. When administered independently, acute carbon monoxide and heat exposure elicited a significant increase in EPO. However, when these interventions were combined, there was interestingly no augmented increase in EPO relative to the two independent exposures, contrary to the authors’ working hypotheses. This null finding raises an intriguing question as to why the EPO response is not augmented, even though in theory the physiological stimulus to increase EPO is greater when both interventions are applied. Relative to practice, this encourages scientists to consider the non‐linear summation of physiological stressors for desired haematological (or other) responses/adaptations. In other words, a greater stressor or stimulus along the oxygen transport cascade may not always equate to greater adaptive response. For translational research involving sports performance, these findings suggest that investigators should strongly consider the utility of added ergogenic interventions relative to pre‐existing training methodologies.

There are putative sex differences in oxygen transport and regulation, ranging from simple differences in oxygen‐carrying capacity and exercising cardiac output, to more nuanced differences in skeletal muscle and mitochondrial oxidative capacity (Ansdell et al., 2020). In this context, the second novel finding of DiMarco et al.’s study addresses potential sex differences in the EPO response to acute carbon monoxide and heat exposure (DiMarco et al., 2024). In brief, investigators found that acute carbon monoxide/heat exposure led to an increase in EPO among females that was not observed in males, suggesting that the acute intervention may be more effective among females. Although important physiological variables were assessed, investigating the exact mechanisms of renal oxygenation and EPO production is exceptionally challenging in human studies, due to the methodological invasiveness necessary for precise measurements (i.e., invasive renal catheterization to measure arterio‐venous oxygen difference). For instance, DiMarco et al. acknowledge potential limitations in renal blood velocity measurements from Doppler ultrasound (inability to confirm a consistent diameter).

Despite these limitations, the study findings evoke several questions and hypotheses. First, it may be valuable to generate ‘dose–response’ curves that quantify the EPO response as a function of carboxyhaemoglobin and core body temperature. Within these curves, some experiments could be used as control trials by measuring fluctuating EPO and sham carbon monoxide and heat exposure. Based on DiMarco et al.’s findings, one may hypothesize that females need a lower ‘dose’ of acute intervention (carbon monoxide/heat exposure) to elicit similar changes in EPO compared to male counterparts. Relative to previous investigations, an additional explanation may be that males require a longer duration of carbon monoxide exposure to elicit a significant EPO response, rather than a simple bolus (Montero & Lundby, 2019). Second, additional exposures (i.e., chronic intervention) may alter the EPO response in a non‐linear way that might also differ between sexes. Lastly, since EPO takes days/weeks to increase circulating red blood cell levels, the next logical step appears to be the quantification of how acute EPO responses to acute carbon monoxide and heat exposure may go on to alter haematological parameters such as total haemoglobin mass, and whether these alterations themselves be different between sexes

Altogether, DiMarco and colleagues' work highlights the considerable mechanistic complexity of systemic and renal oxygen homeostasis following acute carbon monoxide and heat exposure, giving rise to further experimental questions and hypotheses. Beyond the two applied methodologies, the study findings bring forth a provocative notion for physiologists, that even when an individual response is well understood for a given stimulus, the combination of several disparate stimuli may not result in a summative response as anticipated.

## AUTHOR CONTRIBUTIONS

Both authors approved the final version of the manuscript and agreed to be accountable for all aspects of the work in ensuring that questions related to the accuracy or integrity of any part of the work are appropriately investigated and resolved. All persons designated as authors qualify for authorship, and all those who qualify for authorship are listed.

## CONFLICT OF INTEREST

The authors declare no conflicts of interest.

## FUNDING INFORMATION

The presented work was performed in absence of external funding.
